# Recent advances in enzyme-free and enzyme-mediated single-nucleotide variation assay *in vitro*

**DOI:** 10.1093/nsr/nwae118

**Published:** 2024-03-27

**Authors:** Erhu Xiong, Pengfei Liu, Ruijie Deng, Kaixiang Zhang, Ronghua Yang, Jinghong Li

**Affiliations:** Key Laboratory of Chemical Biology & Traditional Chinese Medicine Research, Ministry of Education, College of Chemistry and Chemical Engineering, Hunan Normal University, Changsha 410081, China; Key Laboratory of Chemical Biology & Traditional Chinese Medicine Research, Ministry of Education, College of Chemistry and Chemical Engineering, Hunan Normal University, Changsha 410081, China; College of Biomass Science and Engineering, Healthy Food Evaluation Research Center, Sichuan University, Chengdu 610065, China; School of Pharmaceutical Sciences, Key Laboratory of Targeting Therapy and Diagnosis for Critical Diseases, Zhengzhou University, Zhengzhou 450001, China; Key Laboratory of Chemical Biology & Traditional Chinese Medicine Research, Ministry of Education, College of Chemistry and Chemical Engineering, Hunan Normal University, Changsha 410081, China; Department of Chemistry, Center for Bioanalytical Chemistry, Key Laboratory of Bioorganic Phosphorus Chemistry & Chemical Biology, Tsinghua University, Beijing 100084, China; Beijing Institute of Life Science and Technology, Beijing 102206, China

**Keywords:** single-nucleotide variant, point mutation, *in vitro*, enzyme-free assay, enzyme-mediated assay

## Abstract

Single-nucleotide variants (SNVs) are the most common type variation of sequence alterations at a specific location in the genome, thus involving significant clinical and biological information. The assay of SNVs has engaged great awareness, because many genome-wide association studies demonstrated that SNVs are highly associated with serious human diseases. Moreover, the investigation of SNV expression levels in single cells are capable of visualizing genetic information and revealing the complexity and heterogeneity of single-nucleotide mutation-related diseases. Thus, developing SNV assay approaches *in vitro*, particularly in single cells, is becoming increasingly in demand. In this review, we summarized recent progress in the enzyme-free and enzyme-mediated strategies enabling SNV assay transition from sensing interface to the test tube and single cells, which will potentially delve deeper into the knowledge of SNV functions and disease associations, as well as discovering new pathways to diagnose and treat diseases based on individual genetic profiles. The leap of SNV assay achievements will motivate observation and measurement genetic variations in single cells, even within living organisms, delve into the knowledge of SNV functions and disease associations, as well as open up entirely new avenues in the diagnosis and treatment of diseases based on individual genetic profiles.

## Teaser

This review article delves into current powerful technologies capable of identifying single-nucleotide variants in vitro, which may contribute to the understanding of the complexities of the single-nucleotide variant-related diseases and discovering new ways to diagnose and treat diseases based on individual genetic profiles.

## INTRODUCTION

Single-nucleotide variants (SNVs), also known as single-nucleotide alterations (SNAs) refer to the variation of a single nucleotide that occurs at a specific location in the genome [1]. Both endogenous and exogenous DNA damage can cause the occurrence of SNVs [2]. SNVs are the most common type of sequence alterations at the genomic level, and often hold significant clinical and biological information, thus providing accurate evidence for disease susceptibility and benefit individualized treatment [3,4].

In recent years, many genome-wide association studies have demonstrated that SNVs are highly associated with various human diseases [5,6]. On the one hand, SNVs fall in the coding domain of vital genome that changes the amino acid sequence of encoded protein, thereby affecting the functions of cells and tissues [7]. For example, SNVs in the *C13orf31* (*LACC1*) gene that encodes p.C284R and p.I254V in FAMIN protein, are highly correlated with increased risk for systemic juvenile idiopathic arthritis, leprosy and Crohn's disease [8]. On the other hand, SNVs occurring in some non-coding domains may also influence the physiological activity of cells or tissues’ transcription factor binding, digestion of messenger-ribonucleic acid (mRNA), and more [9]. In addition, some SNVs can serve as predictors to evaluate the risk of tumor metastasis and drug resistance [10]. For instance, the SNVs in the *PIK3CA* gene associated with ∼20% of all breast tumors, have been utilized to predict resistance to trastuzumab treatment, wherein trastuzumab is a widely-used antibody drug for human epidermal growth factor receptor 2 (HER2) treatment [11]. These discoveries may contribute to the study of the essence of gene variation, the identification of new disease-related genes, the development of genetic diagnosis, and the exploration of precise medicine [12]. Given the substantial biological and biomedical implications of SNVs, next-generation sequencing (NGS, also called high-throughput sequencing), a method that can characterize the whole genome sequence information in a high-efficiency fashion, has been extensively employed to discover new SNVs [13] and routinely detect SNVs [14–16]. Nevertheless, the large amount of sequencing data produced by NGS is non-informative wild-type (WT) sequences, which may increase the difficulty of analysis and be of finite worth for routine use [13,17].

**Figure 1.**
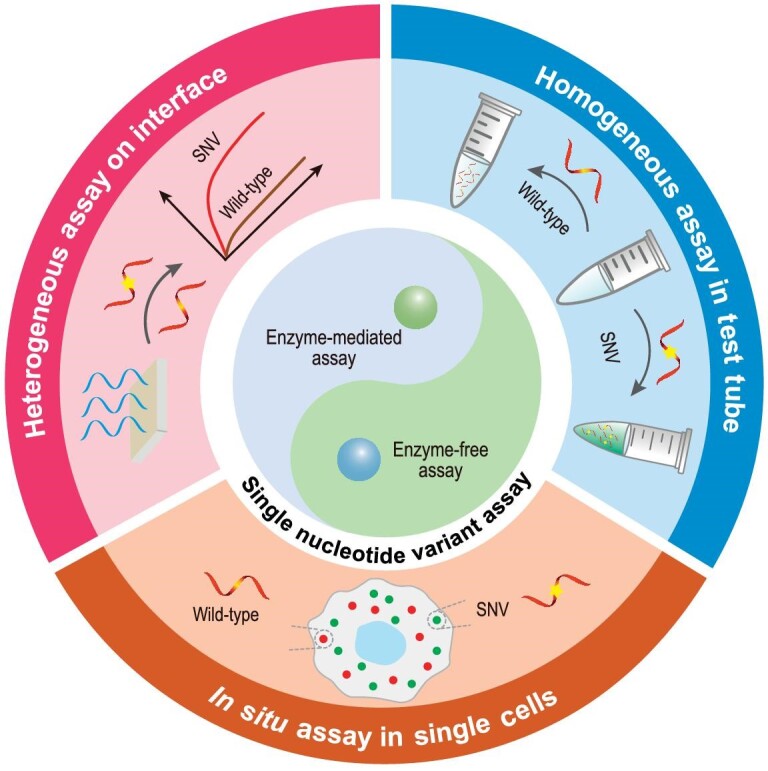
Schematic illustration of enzyme-free and enzyme-mediated SNV assay.

To address this issue, numerous non-sequencing detection strategies have sprung up to achieve simple, fast and cost-effective SNV detection. In this review, we systematically summarize and discuss the recently developed detection strategies for *in vitro* SNV analysis from enzyme-free and enzyme-mediated aspects and, in more detail, divide these strategies into heterogeneous SNV assay, homogeneous SNV assay in the test tube, and *in situ* SNV assay in single cells (Fig. 1). More especially, we will highlight the emerging CRISPR (Clustered Regularly Interspaced Short Palindromic Repeats) technology for SNV detection. In these mentioned sections, some well-known single-nucleotide polymorphism (SNP) sites may be adopted as models for SNV detection. Finally, we will discuss the chance of *in vitro* SNV detection and project some perspectives on future applications. We aim to provide a comprehensive review of developed detection strategies for *in vitro* SNV analysis, and hope that it can contribute to the further developments and applications of this advanced technology.

## DETECTION STRATEGIES FOR *IN VITRO* SNV ASSAY

Considering the complex relationship between SNVs occurrence and disease and biological traits, rapid and efficient identification of specific genes and their mutations can help in disease diagnosis and precision medicine. NGS is a robust method available today for identifying common and rare SNVs. But an average intrinsic error of at least 0.2% makes conventional NGS struggle to report SNVs with very low variant-allele frequency [18]. This problem has been settled by sequence-selective variant enrichment-integrated low-depth sequencing [15,16]. Despite the success of SNV assay by using the NGS platform, a major drawback is that the large amount of sequencing data produced by NGS are non-informative WT sequences, which inevitably makes data processing and analysis complex, time-consuming and high-cost [17,19].

Nowadays, plentiful non-sequencing–based techniques have been successively introduced for *in vitro* SNV detection, such as melting profile analysis, hybridization chain reaction (HCR), toehold-mediated strand displacement (TMSD), programmable DNA molecular computation, locked nucleotide acid (LNA), peptide nucleotide acid (PNA), DNAzyme, polymerase chain reaction (PCR), rolling circle amplification (RCA), CRISPR technology, and so forth. These strategies can be specifically classified into heterogeneous SNV assay, homogeneous SNV assay in the test tube, and *in situ* SNV detection in single cells. In more detail, all of them are further divided into enzyme-free and enzyme-mediated strategies. We will discuss the working principles, merits, and possible limitations of these strategies for *in vitro* SNV assay.

## HETEROGENEOUS SNV ASSAY

For most heterogeneous SNV assay technologies, the three indispensable components include a capture probe to recognize and bind to a target, a transducer interface (such as electrode, microarray, microchip or paper) to immobilize the capture probe, and a signal transducer to obtain detection signals. Recognition and binding events are generally achieved through specific Watson-Crick base-pair interaction or the affinity property of nucleic acid-related enzymes. The immobilization is mainly dependent on covalent interactions including Au-thiol bond, amido bond, and so forth. Signal transducers are usually recruited to evaluate whether the binding event has occurred by recording the signal changes in the local environment.

### Enzyme-free heterogeneous SNV assay

In a general enzyme-free nucleic acid assay, DNA or RNA oligonucleotides can be easily recognized by their complementary strand in a sequence-specific manner. The precise and specific base-pairing endows those fully complementary strands with the capacity to perfectly hybridize with each other. However, a single-base variant in one strand can induce hybridization products that are less energetically favored and thermodynamically stable [20]. In such a situation, the hybridization of immobilized probes with target sequence can be measured by different signal transducers.

Oligonucleotide-based microarray is a heterogeneous analytical tool for large-scale parallel analysis of genetic mutations that works by exploiting the preferential hybridization of the capture probe with target sequence [21]. These microarrays made from different substrates often consist of tens to thousands of individual probe-immobilized reaction areas, thus allowing continuous monitoring of the hybridization between target sequence and its complementary probe. Recently, oligonucleotide-based microarray has been integrated with melting profile analysis for multiplex SNV assay [22,23], which is based on a mechanism whereby a perfectly complementary double-stranded DNA (dsDNA) can withstand a higher melting temperature (*T*_m_) than the one with a single-base mismatch. For example, Rizzi *et al*. developed a giant magneto-resistive (GMR) biosensor array and simultaneously profiled DNA mutations and methylation sites of melanoma cell lines [23]. In this assay workflow, a set of two DNA probes complementary to the WT and mutant-type (MT) sequences were pre-immobilized on the interface of a GMR biosensor array through surface chemistry in order to capture single-stranded DNA-conjugated magnetic nanoparticles (MNP-ssDNA), in which the ssDNA was obtained by a series of treatment procedures of the target DNAs with mutations. After the interface-tethered probes captured the MNP-ssDNA, the magneto-resistive signal (ΔMR) experienced significant change ratio with the continuous increase in temperature. Through melting curve measurement of these DNA hybrids on the array, both mutation and methylation analysis could be determined in a single platform. Nevertheless, if the type of DNA mutations does not intervene in the *T*_m_ change (such as G–C to C–G changes), *T*_m_-based analysis is weakly sensitive to these SNVs due to the negligible discrepancy of the melting profiles [24]. In addition, this approach is highly dependent on accurate command of temperature so as to capture the changes in the binding state of DNA with gradually increasing temperature [25].

**Figure 2.**
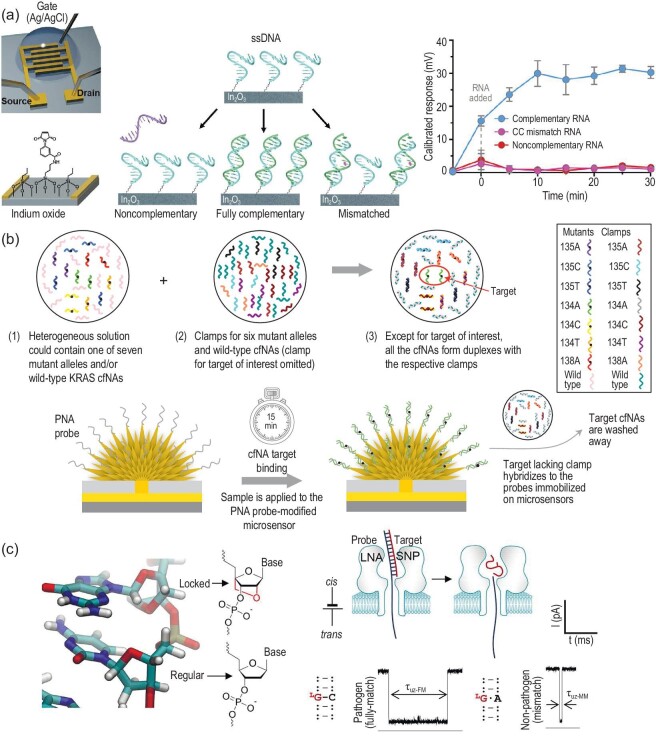
Enzyme-free heterogeneous SNV and SNP assays. (a) Schematic diagram of In_2_O_3_ FET for SNV detection. Reprinted with permission from ref. [26]. (b) Schematic diagram of PNA-assisted electrochemical analysis of mutated cfNAs. Reprinted with permission from ref. [30]. (c) Schematic diagram of LNA-nanopore sensor for SNP assay. Reprinted with permission from ref. [35].

Electronic field effect transistor (FET) is another heterogeneous technology for nucleic acid assay through directly transducing binding events into changes in FET conductance (electronic signals). Due to the merits of fast response time, parallel sensing and high sensitivity, a series of FET-based sensors have been exploited to distinguish genetic mutations [26–28]. For instance, Andrews and Weiss developed an ultrathin-film In_2_O_3_ FET to detect SNV [26]. The representative detection process and mechanism is illustrated in Fig. 2a. The capture probes were immobilized in the semiconducting channels in FETs that obtained a baseline current response. When the fully complementary targets bind to the capture probe, the FET channel conductance experienced an initial increase and remained stable, while a single-base mismatched sequence induced an initial increase and subsequently returned to near baseline with extended time. In this way, the sensing platform successfully distinguished DNA and RNA sequences with single-base mutation. Furthermore, another FET-based SNV assay found that shorter hybridization probes exhibited more superior performance because shorter DNA duplexes are more impressionable to those SNVs [27]. Additionally, through real-time monitoring, the binding kinetics and affinity of the designed shorter hybridization probes and target sequences with various SNV sites, demonstrated that the sequence with SNV sites exhibited slower association and faster disassociation rates compared with the fully complementary sequence. More importantly, the SNV site near the center of the sequence made a more significant impact on the binding kinetics and affinity than those close to the terminal ends because of interaction obstruction, which provides new insight for the rational design of the capture probe.

Except for FET conductance, binding events between immobilized probe and target sequence can also be transduced into electrochemical signals. Electrochemical methods have been applied successfully for point mutations in recent years [29,30]. To further improve detection performance (specificity), researchers recruited synthetic peptide nucleic acid (PNA) as an enhanced capture probe. PNA, a type of nucleic acid analogue that was first proposed by the Nielsen group, utilizes clustered peptide-like N-(2-aminoethyl) glycine units as a linker to attach nucleobases [31]. It can form a more thermally stable duplex with DNA than pure DNA duplex and can benefit from the unique uncharged property of the peptide backbone, endowing PNA-based hybridization probes with higher single-base specificity for SNV discrimination [32]. Based on the property of higher binding affinity with nucleic acid, Das *et al*. proposed a PNA-assisted electrochemical clamp assay to directly detect mutated cell-free nucleic acids (cfNAs) in patient serum samples [30]. Schematic representation of the PNA-assisted electrochemical clamp approach is shown in Fig. 2b. As a proof-of-concept, mutated Kirsten rat sarcoma-2 virus (KRAS), a gene related to several cancers, was selected as the model target. For KRAS 134A mutation detection, a series of clamps for six other mutant alleles and WT sequence were introduced into a solution containing the 134A mutation sequence. After hybridization, all six mutant alleles and WT sequence formed duplexes with their respective clamps except for the 134A mutation sequence. Then, the solution containing the unhybridized 134A mutation sequence was incubated with the PNA probe-modified microchip. As a result, the 134A mutation sequence can effectively hybridize with the probes immobilized on the microchip. Through reading out corresponding electrochemical signals, the mutation sequence can be easily identified. This strategy was further extended to determine mutated circulating tumor DNA against up to a 10 000-fold excess of WT sequences [33].

Nanopore-based sensors provide an ultra-sensitive and label-free detection platform with single-nucleotide sensitivity through monitoring the current signature during the translocation process, and have been broadly investigated to detect SNP and cancer-derived point mutations [34,35]. Interestingly, in a recent study, Tian *et al*. developed a locked nucleic acid (LNA)-enhanced nanopore single-molecule sensor for genetic determination of Shiga toxin producing *Escherichia coli* (STEC) serotype O157: H7, cancer-derived EGFR L858R and KRAS G12D driver mutations (Fig. 2c) [35]. LNA is another artificial oligonucleotide that was first synthesized by Wengel *et al*., wherein the LNA monomers prefer to preorganize in an N-type or 3′-end conformation due to their bicyclic structure, thereby facilitating base stacking and phosphate backbone prearrangement [36].

As the same as PNA, LNA could also form more thermally stable duplexes with its perfectly complementary DNA or RNA than the mismatched sequences. In the nanopore-based mutation detection, a special LNA probe was designed with an overhanging tail and allowed to form a perfectly complementary duplex with WT target sequence and a single-mismatched duplex with MT target, wherein the tail trapped the duplex into the nanopore and promoted the unzipping/translocation process, driven by the voltage. Since the perfectly complementary duplex is more stable than a single-mismatched one, WT and MT sequences could be identified from the prolonged unzipping time. As a result, an ∼10-fold enhancement on SNP discrimination was achieved [35].

Enzyme-free isothermal amplification technologies, which allow rapid and efficient amplification of targets or recognition signals event under simple conditions at constant temperature, have become important nucleic acid testing tools and have been shown to be suitable for the application of highly specific and sensitive SNV assay. Catalyzed hairpin assembly (CHA) is an enzyme-free isothermal reaction, in which single-stranded oligonucleotides catalyze the hybridization of two hairpin probes to produce dsDNA and amplify the signal via target recycling [37]. Liu *et al.* developed a polydiacetylene microtube waveguide platform through the combination of heterogeneous CHA reaction with competitive inhibition. In this platform, the heterogeneous CHA reaction system can preferentially amplify the signal of the WT targets, while the competitive inhibition system will preferentially hybridize with the MT target and inactivate its signal. The strategy provided a highly selective and sensitive platform for precise differentiation of SNVs at lower expression levels [38].

### Enzyme-mediated heterogeneous SNV assay

In addition to the use of complementary probes to realize heterogeneous SNV assay, some nucleic acid-related enzymes have also drawn great awareness as powerful tools for SNV discrimination, such as DNA mismatch repair protein, ligase, DNA polymerase, and so on, for they can easily sense those sequences with SNVs. Besides, the emergence of CRISPR-associated (Cas) enzymes from the CRISPR/Cas system shows a unique recognition mechanism in a crRNA-complementary fashion, representing an exciting new pathway for SNV discrimination. Furthermore, some other enzymes with eminent catalytic capacity against their specific substrates, can also be applied for efficient SNV detection of urease, horseradish peroxidase (HRP), glucose oxidase, and so forth. In this section, we will highlight some representative enzymes in heterogeneous SNV assay together with rapid and efficient detection of infectious disease-associated nucleic acid variations (example of SARS-CoV-2 variants).

**Figure 3.**
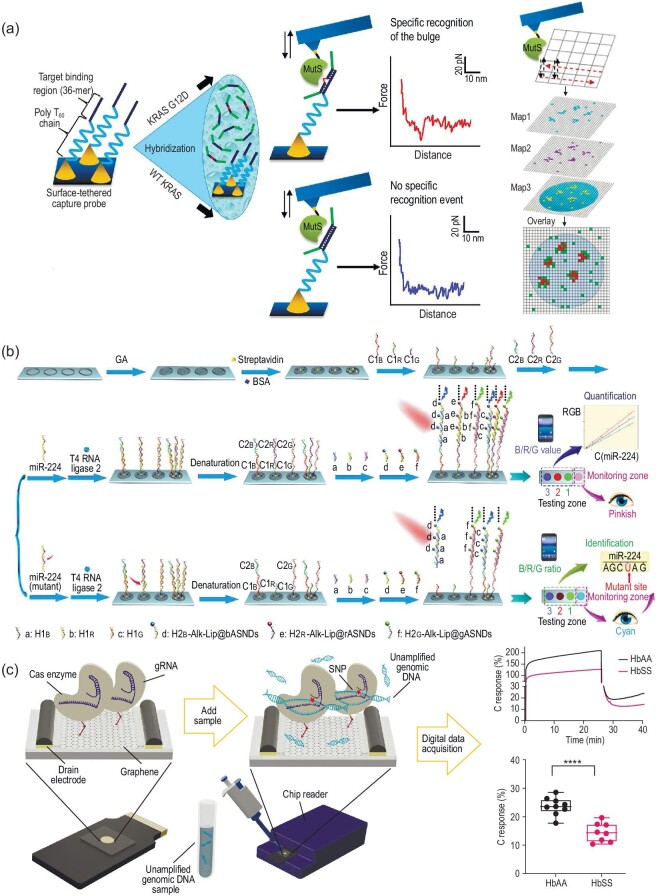
Enzyme-mediated heterogeneous SNV assay. (a) Schematic diagram of MutS protein-mediated F-D-based AFM for KARS G12D mutation assay. Reprinted with permission from ref. [41]. (b) Schematic diagram of electrochemical platforms for miRNA-224 quantification and SNV in miRNA-224 by coupling ligase-mediated HCR with NIR-excited ASNDs. Reprinted with permission from ref. [43]. (c) Schematic diagram of Cas9-immobilized gFET for SNP detection and typical results. Reprinted with permission from ref. [52].

Living organisms have evolved a variety of DNA repair patterns to restrain the occurrence of mutations, including mismatch repair, base excision repair, and nucleotide excision repair [39]. Prior to the DNA repair processes, some particular proteins need to be identified, and then bound to injured or mismatched dsDNA. A representative example is MutS protein, which possesses extremely higher affinity to mismatched DNA duplexes (10- to more than 1500-fold) than to fully complementary base pairs [40]. This feature enables MutS protein as a forceful mismatch-discriminating tool for SNV detection. Based on the unique feature of MutS protein, Kim *et al*. integrated MutS protein with an LNA/DNA chimeric probe to directly analyze the low abundance of targets with SNV site (KRAS G12D mutation) in clinical samples by force-distance (F-D) curve-based atomic force microscopy (AFM) (Fig. 3a) [41]. In this work, MutS protein was employed to bind to the mismatched LNA/DNA-DNA heteroduplexes, in which an LNA/DNA chimeric probe with high affinity was designed to capture the desired target sequence in order to form LNA/DNA-DNA heteroduplexes after a denaturation procedure. And then, through effective capture spot area scanning, force measurement and conscientious analysis, an uppermost detection limit was realized (three copies, 0.006% allele frequency), accompanied by excellent sensitivity/specificity (near to 100%). As a simple extension, this method could identify mismatched or deletion duplexes by up to four bases.

Ligases are a type of significant enzymes with the function of nucleotide ligation, which can recognize mismatched nucleotides at the ligation site [42]. Generally, a circularizable padlock probe or two linear oligonucleotide probes were designed and arranged with a discriminatory base at its terminal (5′- or 3′-terminal), to hybridize with a given target sequence, generating an adjacent nucleotide. Subsequently, the ligase selectively joins with the adjacent nucleotide when the nucleotide at the junction is fully base-paired. Thus, the occurrence (or not) of the ligation reaction can be served as an indicator to determine the presence of SNV [42]. Usually, ligation-based SNV detection has been successfully operated with various amplification strategies, such as HCR [43], PCR [44], and RCA [45], to obtain amplified ligation products and signals. HCR is an isothermal amplification method, in which two hairpins cross-open each other with the assistance of a trigger, thus producing a long and nicked double-helix chain and enabling eminent amplification efficiency [46]. Zhu *et al*. reported handheld electrochemical platforms for miRNA-224 quantification and SNV in miRNA-224 by the combination of ligase-mediated HCR amplification strategy and NIR-excited alkaline-earth sulfide nanodots (ASNDs) [43]. As shown in Fig. 3b, three pairs of probes (C1_R_/C2_R_, C1_G_/C2_G_, and C1_B_/C2_B_) were designed according to miRNA-224 sequence, wherein C1_R_, C1_G_, and C1_B_ were pre-immobilized on the interface of the refitted diagnosis kits in three parallel regions, while C2_R_, C2_G_, and C2_B_ contain different overhanging sequences at the 3′ terminal. In the presence of miRNA-224, three pairs of probes can hybridize with miRNA-224, respectively, and form duplexes without nicks upon the activity of T4 RNA Ligase 2. Then, the overhanging sequences acted as the initiator of individual HCRs and generated a large amount of ASND-labeled HCR products, thus generating strong fluorescent signals. Consequently, the handheld platform can achieve quantitative detection of miRNA-224. Even more, benefiting from the bright fluorescence and tunable emission wavelength of NIR-excited ASNDs, the SNV in miRNA-224 can lead to remarkable changes of fluorescent brightness and color in RGB channels, thereby enabling on-site distinguishing of different SNVs in miRNA-224.

Additionally, Cas enzymes are another momentous tool for SNV discrimination. CRISPR/Cas systems, which are derived from the adaptive immune system of archaea [47], have been revolutionized for genome editing, genomic loci imaging, and nucleic acid detection in a user-defined manner [48,49]. Cas9, a typical example of Cas enzymes, can recognize target sequence near short 5′-NGG-3′ protospacer adjacent motif (PAM), then bind and induce double-strand break (DSB) under the guidance of its guide RNA (gRNA). The gRNA is composed of a CRISPR RNA (crRNA) and a *trans*-activating RNA (tracrRNA), and can be further engineered as a single RNA chimera [50]. It is reported that Cas9/gRNA complex exhibits high specificity toward base mismatch within the first few nucleotides in the so-called seed sequence [51]. This unique mechanism has been adopted for SNP detection in unamplified genomic DNA through Cas9 (or dead Cas9, dCas9)-immobilized gFET (termed SNP-Chip) by Aran's group (Fig. 3c) [52]. Results showed that SNP-chip can discriminate homozygous and heterozygous target sequences differing by a single nucleotide within 1 h. This strategy not only can be extended for the high-efficiency discrimination of SNP genotyping, but also contributes to monitoring the efficacy of the Cas9/gRNA complex.

**Figure 4.**
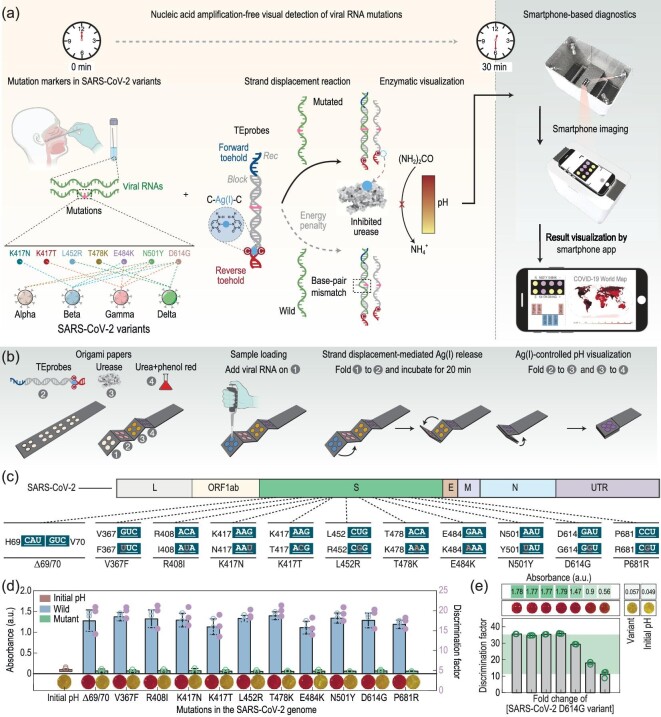
Urease-mediated paper-based colorimetric detection of SARS-CoV-2 variants. (a) Schematic diagram of SARS-CoV-2 variants discrimination with MARVE. (b) Workflow of the MARVE. (c and d) SARS-CoV-2 RNA mutations and visual detection of these mutations. (e) Evaluation discriminative capacity of MARVE by using SARS-CoV-2 D614G variants upon the addition of excess wild SARS-CoV-2. Reprinted with permission from ref. [56].

Similar to identifying human genetic mutations, detecting infectious disease-associated nucleic acid variations is essential for personalized and preventative medicine. For example, emergence of the novel variants of coronavirus (COVID-19) has highlighted the requirement for rapid, flexible diagnostic assays that are easily deployable, manufacturable, and adaptable to new infectious agents once new genomes are sequenced and identified [53–55]. To this end, some excellent studies have been done to rapidly resolve mutations in SARS-CoV-2 for discriminating its variants [56–58]. For example, Yang *et al*. designed a rapid and highly sensitive DNAzyme-based detection system, REVEALR (RNA-encoded viral nucleic acid analytic reporter), for SARS CoV-2 variant identification. The REVEALR system identified the correct variant [Wuhan-Hu-1, alpha (B.1.1.7), gamma (P.1), epsilon (B.1.427/9), delta (B.1.617.2), and omicron (B.1.1.529)] with 100% accuracy [57]. Chen *et al*. reported a CAVRED (CRISPR-based amplification-free viral RNA electrical detection) platform by combining CRISPR technology with FET arrays, and achieved simultaneous profiling of nine critical RNA mutations associated with SARS-CoV-2 variants of concern at single-nucleotide resolution [58]. Zhang *et al*. pursued a paper-based strategy, named MARVE (short for multiplexed, nucleic-acid-amplification-free, single-nucleotide-resolved viral evolution), which involves a common pH indicator, phenol red that experiences a considerable color change (from red to yellow) when pH decreases (Fig. 4) [56]. A collection of toehold exchange DNA probes (TEprobes) consisting of a pair of terminal single-stranded overhangs, termed the forward and reverse toehold, have been designed to recognize SARS-CoV-2 variants including Alpha, Beta, Gamma and Delta, wherein a cytosine-cytosine (C : C) mismatch is introduced in the TEprobes to selectively incorporate Ag⁺ to form C-Ag⁺-C complexes due to a strong base-metal cation interaction [59]. Competitive binding of viral RNA with the Rec strand in TEprobes, promoted by the forward toehold, disrupts the reverse toehold and releases Ag(I) ions. In the presence of viral RNA, the clamped Ag⁺ is released from the TEprobes, and subsequently prevents urease from hydrolyzing urea to produce the weak base ammonia [60], thus resulting in lower pH conditions and a considerable color change (Fig. 4a and b).

In this way, SARS-CoV-2 mutations and variants could be easily distinguished and visualized by the naked eye using pH indicators (Fig. 4c and d). Additionally, MARVE showed eminent discriminative capacity toward the SARS-CoV-2 D614G variant even in 50-fold molar excess WT SARS-CoV-2 (Fig. 4e). The authors further demonstrated that MARVE permitted simultaneous detection of different SARS-CoV-2 variants by adopting multiple sample-loading sites via naked eye or smartphone-based image processing by setting a threshold GAR value. This work provides a portable and user-friendly technology for the diagnosis and screening of infectious disease-associated nucleic acid variations and other threatening pathogens.

## HOMOGENEOUS SNV ASSAYS IN THE TEST TUBE

Different from heterogeneous SNV assay, the capture probes in homogeneous systems can diffuse in a free manner, which improves the probability of probe-target binding events to some extent, thus facilitating diagnostic applications. The binding events can be easily converted into fluorescence, visual signal, and so on. We also discuss strategies for homogeneous SNV assay in the test tube from enzyme-free and enzyme-mediated patterns.

### Enzyme-free homogeneous SNV assay

**Figure 5.**
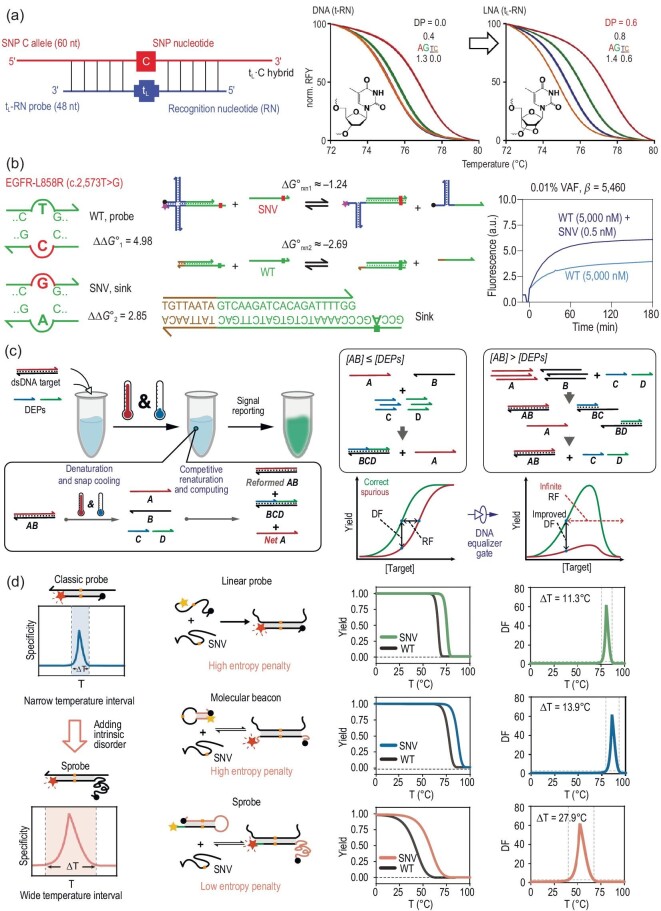
Enzyme-free homogeneous SNV assay. (a) Schematic diagram of t_L_-based HRM for SNV detection. Reprinted with permission from ref. [61]. (b) Schematic diagram of simulation-guided DNA probe design for ultra-specific SNV assay. Reprinted with permission from ref. [66]. (c) Schematic diagram of DNA equalizer gate for expanding the SNV detection window. Reprinted with permission from ref. [70]. (d) Schematic diagram of coding intrinsic disorder into DNA hybridization probes for the discrimination of SNVs over wide and tunable temperature ranges. Reprinted with permission from ref. [73].

High-resolution melting (HRM), which is based on liquid-phase melting profile, has seen widespread use to scan of cancer-related gene mutations. In a typical HRM, the DNA probe can only discriminate one perfectly matched allele from the other mismatched alleles. To improve the discrimination capacity, Obliosca *et al*. designed a simple melting probe embedded with a single locked thymidine monomer (t_L_), which can reliably discriminate all four SNP alleles by four different *T*_m_ in a single experiment (Fig. 5a) [61]. This phenomenon can be attributed to the involvement of LNA which decreased *T*_m_ of the t_L_·C mismatched duplex but increased or retained *T*_m_ of the other three hybrids.

TMSD reaction based on competitive hybridization reaction mechanisms can also be adopted for homogeneous SNV detection. In a conventional TMSD reaction, a longer single-stranded sequence can land on a prehybridized duplex through an overhanging domain (often termed toehold), subsequently experience a branch migration process and release a shorter strand from the duplex, and finally form a more stable duplex, exhibiting a great potential for SNV analysis due to the fact that sequences containing SNV site(s) showed a slower reaction kinetics [62]. It is noteworthy that this approach is insufficient to distinguish those SNV sites far away from the toehold domain because of negligible reaction kinetics difference. To address this issue, a two-step TMSD reaction has been developed [63]. In this method, a helper strand was recruited to initiate the next competitive hybridization reaction after the first strand displacement reaction in order to achieve the overall reaction. The two-step TMSD reaction showed much higher specificity for SNV discrimination. Although great efforts have been made for the rational design of thermodynamics-guided probes and empirical optimization of the reaction conditions, only limited improvements in differential binding affinity have been obtained [64,65]. The above-mentioned hybridization-based SNV detection approaches may still suffer from unintended binding due to the similarity of mutated and WT sequences. To address this issue, Zhang *et al.* reported simulation-informed competitive compositions which include an SNV-specific probe and a WT-specific sink molecule, by constructing a kinetic reaction model to predict the optimal combination of thermodynamic parameters [66]. By using a simulation-guided X-probe, competitive compositions achieved 200- and 3 000-fold (median 890) higher specificity for 44 cancer-related SNVs, even in the presence of 10 000-fold excess of WT sequences (Fig. 5b). Moreover, the authors further extended the simulation-guided design approach to detect low variant allele frequency sequences of PCR-amplified human genomic DNA, and artificial RNA sequences. This study may motivate the rational design of molecular reagents and diagnostics.

DNA molecular computation has been well characterized to be a powerful tool for carrying out complicated computation on disparate substrates toward diverse applications, given that DNA molecules can simultaneously react with various other molecules, logically process information, and provide corresponding results in a programmable way [67,68]. Some rational-designed DNA molecular computation systems have been adopted to generate diagnostic information of cancer screening and respiratory infections, of which multiplex biomarkers recognition are integrated with logical information processing to motivate fast, accurate, and economic diagnostic mode [69]. For example, Wang *et al*. developed a DNA equalizer gate (DEG) approach by coupling DNA-based computation with a series of simulation-guided hybridization probes, which can dramatically expand detection windows for SNV detection through a user-defined transformation of the quantitative relationship between the detection signal and target concentrations (Fig. 5c) [70]. In addition, Han and coworkers proposed a switching circuit (SC)-based DNA computational system, which enables multiple detection and logical analysis of SNPs in clinical blood samples and has 100% consistency with NGS [71].

DNA hybridization probes are commonly used tools for identifying clinically important SNVs, and the SNV identification mainly depends on the DNA hybridization probes or primers, which respond to the subtle difference of free energy between an SNV and its WT sequence. However, conventional linear hybridization probes face some challenges, such as low specificity in SNV assay at low temperatures and low affinity in binding folded oligonucleotides. Recently, to address these issues, Mueller *et al.* developed a multicomponent hybridization probe, which employed two target binding arms to tightly bind and unwind a folded target sequence, and two sequence-specific strands to bind the target and stem-loop molecular beacon (MB) probe to open it to generate fluorescence signals, enabling it to differentiate SNVs in folded analytes in the temperature range of 5–38°C [72]. Further, through investigation of the thermodynamic basis of the narrow temperature intervals for the traditional linear ssDNA hybridization probes and MB probes, Guo *et al.* found that the high entropy penalty was the major obstacle to the effective identification of SNVs at a wide temperature range. Guided by this finding, they designed hybridization probes (Sprobes) with high intrinsic disorders to compensate for the entropy penalty, resulting in wide and tunable temperature ranges for identifying SNVs in clinical samples [73].

### Enzyme-mediated homogeneous SNV assay

**Figure 6.**
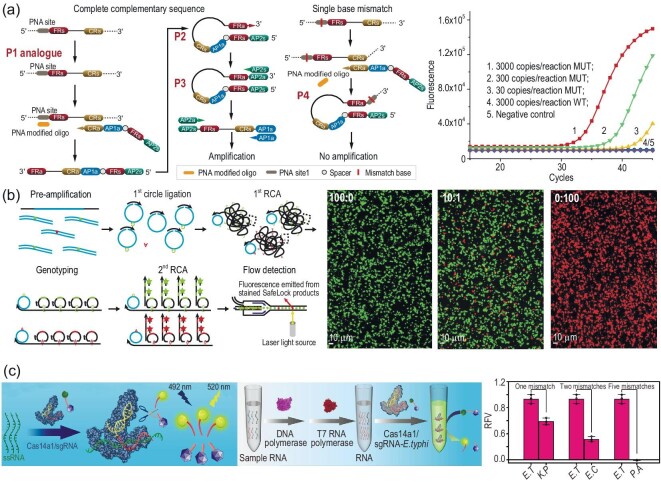
Enzyme-mediated homogeneous SNV assay. (a) Schematic diagram of STEM-PCR for single-base mutation detection. Reprinted with permission from ref. [76]. (b) Schematic diagram of superRCA mutation detection assays [78]. (c) Schematic diagram of ssRNA-activated Cas14a1 for mutations discrimination. Reprinted with permission from ref. [85].

DNA polymerase is a kind of nucleic acid-related enzyme due to its unique mechanism of specifically extending a short primer along its complementary DNA template with the addition of deoxynucleotide triphosphates (dNTPs), which can also be applied for SNV detection [74]. The mechanism employs a pre-designed primer to hybridize with annealed PCR-amplified DNA fragments, generating a primer-SNV site-contained template duplex. Subsequently, DNA polymerase is introduced to incorporate fluorophore-labelled 2′,3′-dideoxynucleotide triphosphate to the 3′-terminal of the primer. When the dideoxynucleotide is incorporated on the primer, the elongation will stop because of a lack of 3′-OH group in dideoxynucleotide, which is extremely significant for polymerase-mediated extension. By analyzing the extension products using electrophoresis or fluorescence, SNV can be easily identified. The analysis performance toward site-specific mutation can be further enhanced by allele-specific PCR (AS-PCR), which recruits a specific AS primer containing nucleotide substitution at its 3′ terminal, allowing preferential amplification of target sequences with SNV sites [75]. Very recently, Gu *et al.* developed a specific terminal mediated polymerase chain reaction (STEM-PCR) to discriminate nucleic acid modifications (site-specific methylation and co-methylation) and single-base mutations with prominent specificity and sensitivity (Fig. 6a) [76]. This strategy depends on the tailored-designed foldable primer (TFP) which is extended along the target sequences to obtain the desired molecular construction with specific terminal domains, allowing self-folding and availing PCR primer binding for subsequent amplification. By using the L858R single-base mutation in EGFR as the model target, the PNA-implemented STEM-PCR achieved a superior sensitivity of 30 copies/reaction, and eminent specificity with 100-fold concentration of WT sequence. In such a case, different mutations could be realized through designing corresponding PNA blockers.

RCA reaction is another enzymatic reaction and acts as a powerful tool for homogeneous nucleic acid amplifications [77]. In RCA reactions, the discriminatory base at both ends of the circularizable padlock probe can form a junction in the presence of target nucleic acids of interest, and the ligase will connect the adjacent nucleotides when the nucleotides are fully base-paired. After ligation, DNA or RNA polymerase extends the strand along the circular padlock probe template, generating long ssDNA or ssRNA strands with tandem repeats. This strategy has been widely used for SNV assay. For example, Chen *et al*. described an approach, termed superRCA, for ultrasensitive and precise quantitation of ultralow frequency point mutations from malignant cells of acute myeloid leukemia (AML) patients [78]. As shown in Fig. 6b, at first, the mutated sequences from patient samples were enriched, and pre-amplified by PCR. Then, the PCR amplification products were converted to circularizable DNA, followed by the first circles of RCA. Afterward, padlock probes specific for MT or WT sequences were utilized to bind to first-generation RCA products with excellent specificity, followed by the second circles of RCA, generating plentiful large DNA clusters referred to as superRCA products. By extra addition of different fluorophore-labeled probes, the MT- and WT-specific individual superRCA products could be easily analyzed through standard flow cytometry or microscopy. In such a case, the superRCA approach enables precise detection of very rare SNV sequences up to a 100 000-fold excess of WT sequences, even SNVs in challenging sequences such as GC-rich regions.

Exponential amplification reaction (EXPAR) is an isothermal enzymatic amplification reaction, which includes a DNA template and two enzymatic reactions to obtain exponential amplification of correct targets and exhibits a high amplification efficiency (10^6^–10^8^) within 30 min [79]. While the single-base mismatch between the primer (3′-end) and template will severely inhibit the amplification efficiency, this makes it attractive for the sensitive assay of nucleic acids [80,81]. Long *et al.* proposed a triple-recognition strategy, which promotes aligner-mediated cleavage-triggered EXPAR for the specific SNV assay [82]. In the study, the authors designed a hairpin-shaped DNA aligner with two side arms, which are complementary to the MT strand with a G-mutation (SNV) and WT strand. After hybridization, it would be cleaved to generate ssDNA by endonuclease with different cleavage efficiency due to the presence of G-mutations. The produced ssDNA with G at the 3′-terminal from the MT strand can bind to the EXPAR template and extended by DNA polymerase to yield dsDNA, while no ssDNA from the WT strand was generated to initiate EXPAR. The platform exhibited a reliable discrimination of 0.1% SNV in the WT sequence, which is lower than that of AS-PCR for SNV assay.

Except for above-mentioned Cas9 effector, some Cas effectors such as Cas12, Cas13, and Cas14, can not only recognize and bind to the target of interest in a crRNA-complementary fashion, but also unleash non-specific cleavage events (called *trans*-cleavage or collateral effect), which can be utilized to construct signal converter for amplified SNV detection [49]. For example, Shi *et al.* developed an autocatalysis-driven feedback amplification network, namely, CRISPR-Cas-only amplification network (CONAN), and investigated its analytical performance by using E545K mutant (a single 1633G > A mutation) in PIK3CA as the SNV model, achieving a low detectable concentration of 5 aM [83]. Gootenberg *et al*. reported a molecular detection platform called SHERLOCK (Specific High Sensitivity Enzymatic Reporter UnLOCKing) to discriminate specific strains of Zika and Dengue virus with single-base specificity [84]. In addition, Wei *et al.* discovered that the CRISPR-Cas14a1 system can also unleash *trans*-cleavage activity on ssDNA when binding to target RNA (Fig. 6c), and the Cas14a1 protein showed more outstanding specificity toward RNA point mutations than DNA [85]. Based on this finding, we constructed a Cas14a1-based RNA-activated detection platform, termed ATCas-RNA (Amplification, Transcription, Cas14a1-based RNA-activated trans ssDNA cleavage) for detection of a series of pathogens in milk samples with 100% accuracy.

## *IN SITU* SNV ASSAY IN SINGLE CELLS

Compared to heterogeneous and homogeneous SNV analysis, *in situ* SNV detection in single cells is able to visualize genetic information so as to better reveal the complexity and heterogeneity of mutation-related diseases [86,87]. One example is to characterize the complexity and heterogeneity of disease-related mutations, which is reflected at the transcriptomic, mitochondrial DNA and nuclear genome levels. It is becoming gradually clear that a disease is often composed of a series of individually distinguishing pathologies, and shows remarkable heterogeneity among different cell subtypes. This complexity and heterogeneity have been increasingly considered as the major reason of therapeutic failure and disease recurrence. Therefore, spatial and temporal knowledge of the abundance and contribution of these mutations may contribute to identify cancer subtypes, resolve the mechanism of gene regulation, explore patient-specific medicine protocols, even infer cancer evolution paths and discover new disease-related genes [88,89]. Besides, *in situ* imaging can be adopted to evaluate treatment outcomes and disease recurrence through detecting those selective removals of mutations [90]. However, complicated cell components in the local environment make it difficult for *in situ* SNV imaging to achieve desired efficiency and accuracy. To address this issue, great efforts have been devoted to exploring appropriate methods that can realize SNV imaging *in situ* in single cells. Major methods for *in situ* SNV detection in single cells depend on fluorescence *in situ* hybridization (FISH), which can be classified into enzyme-free and enzyme-mediated FISH. We will discuss these technologies in detail in the next section.

### Enzyme-free FISH for *in situ* SNV assay in single cells

**Figure 7.**
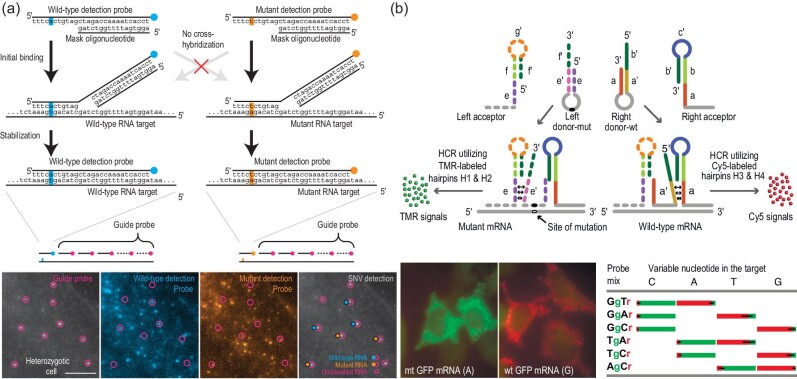
Enzyme-free FISH for *in situ* SNV assay in single cells. (a) Schematic diagram of toehold-mediated FISH-based SNV imaging. Reprinted with permission from ref. [95]. (b) Schematic diagram of HCR-based amp-FISH for WT and MT RNA discrimination by using hairpin probes. Reprinted with permission from ref. [96].

FISH technology has been employed to pinpoint target sequences in single cells for a long period of time and is regarded as a molecular cytogenetic technique to visualize specific target sequences, which play crucial roles in biological and biomedical research. In the FISH-based imaging process, a large number of fluorescent-labeled probes are used to hybridize with annealed DNAs or RNAs to obtain enhanced signals. Later, some modified FISH have been proposed to improve the imaging performance (sensitivity, specificity and high-throughput capability) of FISH, including single-molecule FISH (smFISH) [91], sequential FISH (seqFISH) [92], multiplexed error-robust FISH (MERFISH) [93]. However, a common drawback of the above-mentioned strategies is unsuitability for targeting very small sequence alterations, such as SNVs [94], because single fluorescent-labeled probes may induce undesired off-target signals. To address this issue, Levesque *et al*. explored a toehold-mediated FISH-based imaging approach by using a masked fluorescent-labeled probe to target sequences close to the SNV site (Fig. 7a), in which the SNV detection probe is partly masked by a shorter ‘mask’ strand, producing a short overhanging domain [95]. Only when specific binding occurs, the mask strand is liberated via a strand displacement mechanism, generating a stable detection probe-target RNA duplex. The short overhanging domain serves as the toehold for specific binding of target RNA, and possesses the capacity to discriminate single-base mismatch, thus enabling a very low false-positive binding event. In such a case, this approach realized high-efficiency visualization of MT and WT RNA transcripts with single-nucleotide resolution and further revealed allele-specific expression in single cells. Recently, this approach was repurposed to directly image adenosine-to-inosine RNA editing events with single-nucleotide resolution *in situ*.

The employment of a single probe to direct target sequences with mutations is always constrained by the faint resulting signals. HCR has engaged great awareness due to its eminent amplification efficacy that can considerably increase signal strength and has been utilized to locally amplify signals near the SNV site. For instance, Tyagi's group described a high-fidelity amplified FISH (amp-FISH) to image sparsely expressed MT and WT mRNAs with single-nucleotide resolution (Fig. 7b) [96]. The well-designed probes, that is, two pairs of amp-FISH probes, can only generate amplified signals in a target-dependent manner. Consequently, the two pairs of amp-FISH probes generated distinct *in situ* signals with differentiable colors. It can be seen that HCR provides great promise for *in situ* SNV imaging in single cells due to its moderate experimental conditions and enzyme-free property.

### Enzyme-mediated FISH for *in situ* SNV assay in single cells

**Figure 8.**
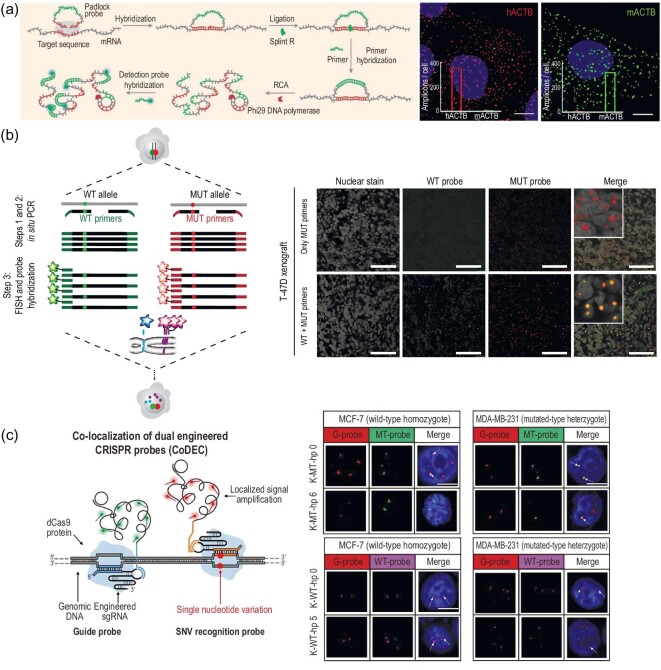
Enzyme-mediated FISH for *in situ* SNV detection in single cells. (a) Schematic diagram of the direct imaging of mRNA by target RNA-triggered RCA. Reprinted with permission from ref. [99]. (b) Schematic diagram of *in situ* PCR based STAR-FISH protocol for SNV identification in nuclear genome. Scale bars: 75 μm. Reprinted with permission from ref. [101]. (c) Schematic diagram of CoDEC probes for SNV imaging in the nuclear genome. Reprinted with permission from ref. [103].

Since the pioneering work on localized DNA imaging of *in situ* RCA, this strategy has been in widespread use to analyze a variety of DNAs/RNAs. Typically, prior to RCA reaction, converting target sequences into a detectable ssDNA is often needed by combination with different strategies according to types of targets, such as a reverse transcription step for RNA analysis. Then, the ligase catalyzes the ligation of circularizable padlock probes upon perfect hybridization with detectable ssDNA. After the amplification process, a localized rolling circle product is generated near the SNV site. Ultimately, fluorophore-labeled detection probes can hybridize with the products, thus realizing the output of imaging signals. By adopting this strategy, Larsson *et al*. achieved the *in situ* SNV discrimination of transcripts (human and mouse β-actin mRNA differing by a single base), and further successfully identified highly related transcripts from mouse embryo tissue, that is, skeletal muscle α1-actin (Acta1) and cytoplasmic β-actin (Actb) transcripts [94]. However, the introduction of a reverse transcription step may lead to a variation in mRNA quantification, thereby embarrassing the detection procedure [97,98]. To overcome this limitation, recently, Li *et al.* proposed a direct mRNA detection method, termed target RNA-initiated RCA (Fig. 8a), without the reverse transcription step [99]. To this end, an extra short primer was recruited to perform *in situ* RCA instead of reverse transcription product. This method enables high-efficiency mRNA imaging with single-nucleotide and near-single-molecule resolution in single cells, providing a great potential in profiling gene expression and genotyping at single-cell level. Despite the success, the *in-situ* ligation of circularizable padlock probes may suffer from topological constraints and the amplification reaction may be hampered by some binding proteins. To solve this problem, Jiang *et al*. skillfully integrated ligation-mediated discrimination with branched HCR for amplified genotyping point mutation of individual mRNAs [100].

As an alternative to RCA-based *in situ* SNV imaging methods, PCR has been applied to amplified image gene sequences for many years. The advances of *in situ* PCR render tremendous potential for *in situ* SNV imaging in fixed cells. In general, *in situ* PCR uses the fixed cells as miniature vessels, in which amplification reactions can effectively take place profiting from the localization of amplicons. The cells are fixed gently to avoid detriment of the cellular structure, and treated with paraformaldehyde to be made permeable, so that PCR reagents can easily enter the cells for localized amplification. After that, the majority of PCR products remain in the fixed cells, thus achieving the *in-situ* imaging of specific targets. Based on this, Polyak *et al.* described a specific-to-allele PCR-FISH (termed STAR-FISH), which can simultaneously detect SNVs and copy number variations at the single-cell level (Fig. 8b) [101]. They utilized STAR-FISH to evaluate the clinical influence of changes in intra-tumoral heterogeneity for HER2 (ERBB2) amplification and the PIK3CA mutation within HER2-positive breast cancer subjected to neoadjuvant therapy, thus obtaining detailed information of spatial distribution characteristics and cancer cells’ subpopulations. Results showed that chemotherapy tended to select those cells containing PIK3CA mutation and participate in the modulation of genetic diversity. This finding indicates that the usage of *in situ* methods may promote more high-efficiency therapies toward heterogeneous tumors. Despite its great potential in tissue section analysis, an insurmountable obstacle of *in situ* PCR is the demand of repeated heating and cooling procedures, which inevitably destroy the integrity of genomic structures and are unsuitable for precise analysis of genomic localization at the subcellular level.

Generally, cleavage-deficient Cas9 (dCas9) is often employed, which only retains full DNA recognition and binding ability to realize precisely specific imaging of genomic locus in cells. For example, Zhang *et al.* proposed a CRISPR/Cas9-assisted proximity ligation assay (CasPLA) to directly discriminate SNV in mitochondria DNA [102]. By integrating simultaneous binding events of dual dCas9/gRNA probes with localized amplification of PLA, CasPLA can clearly reveal the spatial localization of SNVs in the ND4 gene in mitochondria DNA at single-molecule resolution *in situ* in single cells. A major limitation of CasPLA is that the PLA can only occur upon adjacent binding of the two dCas9/gRNA probes. Once the two probes are far away from each other, no PLA occurs. To address this issue, recently, Liang *et al.* developed an improved strategy termed colocalization of dual-engineered CRISPR probes (CoDEC) for visualizing SNVs in the nuclear genome of mammalian cells (Fig. 8c) [103]. In this strategy, an engineered gRNA with a hairpin structure to improve on-target capacity, and a localized RCA to produce bright fluorescence signals at the binding site, were employed. In such a case, CoDEC performed higher on-target detection efficiency and simultaneously visualized WT and MT genes in single cells. Despite the substantial advances, Cas9-based SNV imaging strategies are still constrained from the demand of a PAM sequence [104], thereby restricting the targetable genomic loci. Excitingly, some rationally engineered *Sp*Cas9 (obtained from *Streptococcus pyogenes*) variants such as SpCas9-NG capable of NG PAM recognition [105], and near-PAMless SpRY [106], make it possible to visualize previously inaccessible disease-relevant SNVs. For CRISPR/Cas9-based FISH methods combining with nucleic acid amplification or not, however, in principle, is ineluctably accompanied by the binding of non-specific probes to cellular structures due to the binding dependence of fluorophore-labeled probes. Non-specific probe binding results in high background and signal gain attenuation, requiring a rigorous washing process to mitigate the background. To solve this problem, based on ligase-assisted transcription amplification, Xia *et al.* proposed a light-up strategy for high-contrast SNV imaging in cells without the washing procedure. In this strategy, a light-up RNA aptamer was adopted as a reporter to eliminate the non-specific probe binding and washing process, enabling a two-fold increase in signal gain compared to that employing the FISH method [107].

## CONLCUSIONS AND PERSPECTIVES

In view of the complex relationship of SNV occurrence with diseases and biological traits, accurate and effective identification of specific genes and their mutations is conducive to disease diagnosis and precision medicine. In this review, we have summarized powerful technologies capable of identifying SNVs *in vitro*. These strategies have been classified into three major patterns: heterogeneous SNV assay, homogeneous SNV assay in the tube, and *in situ* SNV detection in single cells. These strategies have been further expanded from enzyme-free and enzyme-mediated aspects, and the detailed mechanism of each approach has been elucidated. Enzyme-free SNV assay mainly involves the use of reaction between nucleic acid strands, and a small majority of particular fluorescent materials, while enzyme-mediated SNV assay mainly relies on DNA mismatch repair protein, ligase, polymerase, urease, and Cas enzymes. We sincerely hope that this review will be conducive to a better understanding of the importance and complexity of SNVs in biological systems, the developments of SNV assay *in vitro*, even *in vivo*, as well as the pros and cons of these enzyme-free and enzyme-mediated strategies for SNV assay (Table 1).

**Table 1.** Summary of the main enzyme-free and enzyme-mediated strategies for SNV assay.


Types	Strategies/technologies	Application models	Advantages	Limitations	Ref.

Enzyme-free SNV assay	Oligonucleotide-based microarray/Melting profile analysis	Heterogeneous assay	• High sensitivity • High throughput analysis	• Complex probe immobilizations • Dependence on accurately commanded temperature	[22,23]
	Electronic field effect transistor	Heterogeneous assay	• High sensitivity and specificity • Real-time monitoring • Label-free assay	• Complex fabrication process • High production cost	[26]
	Nanopore analysis/LNA	Heterogeneous assay	• High sensitivity • Low cost • Portability	• Unsuitable for ultrashort nucleic acid sequence • Expensive LNA probe synthesis • High background noise	[35]
	High-resolution melting/LNA	Homogeneous assay	• High sensitivity and specificity • Simultaneous discrimination of four SNP alleles	• Expensive LNA probe synthesis • Difficult to distinguish more heterozygous samples	[61]
	Multicomponent/Entropy-compensate hybridization probes	Homogeneous assay	• Wide temperature intervals • High specificity and sensitivity	• Precise design and adjustment	[72,73]
	Toehold-mediated FISH	Homogeneous assay	• High specificity • High-efficiency visualization	• Washing process • Require optimization of toehold length	[95]
	HCR-based amp-FISH	Homogeneous assay	• High specificity and specificity • High signal output efficiency	• Washing process • Require optimized hairpin probe	[96]
Enzyme-mediated SNV assay	Paper-based colorimetric detection/MARVE	Heterogeneous assay	• Nucleic acid amplification-free and visualized assay • Simple probe design • Low instrument requirement	• Lower sensitivity than RT-qPCR • Unsuitable for SNV assay in high secondary structure	[56]
	STEM-PCR/PNA	Homogeneous assay	• High sensitivity and specificity • No-cross reaction	• Complex primer design and optimization • Expensive PNA probe synthesis	[76]
	SuperRCA	Homogeneous assay	• High sensitivity and specificity • Suitable for routine use	• Complex experimental process • Multiple-step reaction	[78]
	EXPAR	Homogeneous assay	• Isothermal amplification • High amplification efficiency • Rapid and sensitive assay	• High background noise • Multi-enzyme reaction system	[82]
	FISH/RCA/CasPLA	Homogeneous assay	• Single-molecule resolution • Signal amplification	• Require adjacent binding of dCas9/gRNA probes • Require PAM site • Washing process	[102]
	FISH/RCA/CoDEC	Homogeneous assay	• High on-target detection efficiency • Simultaneously visualized WT and MT genes	• Require PAM site • Washing process	[103]
	Light-up visualized strategy	Homogeneous assay	• Label-free probe • High specificity • Wash-free assay	• Require ligation reaction • Require transcription amplification	[107]

Types Strategies/technologies Application model Advantages Limitations Ref.

Despite tremendous success achieved in SNV assay *in vitro*, some problems and challenges still remain. For most heterogeneous SNV detection strategies, the probe-target binding events, occurring on the surface of a sensor, would be inevitably hampered by the steric hindrance of immobilized probes. Besides, the immobilization of probes on the sensor interface is a laborious procedure often accompanied by non-specific adsorption, which may also affect the detection. Thus, rational regulating density of immobilized probes, and the involvement of surfactant, to some extent, may be beneficial to decrease steric hindrance, and eliminate non-specific adsorption, thus improving the detection performance. For homogeneous SNV assay in the tube, most tests were carried out in well-controlled buffer systems, not in biological fluids or cells. Therefore, the detection probes may become unstable because the complicated matrix in human serum or plasma may have a significant impact on the assay due to the lack of separation processes. An alternative method is to further optimize or modify the detection protocol and carefully evaluate the complicated matrix. In addition, given the inherently low spatial resolutions of homogeneous assays, it is a great challenge to adapt them to the simultaneous discrimination of multiplex SNVs. Introducing the homogeneous assay to picolitre-scale droplets or single-molecule fluorescence imaging may meet the demand of multiplex SNVs. For *in situ* SNV detection in single cells, a key obstacle is to visualize and distinguish genomic sequences with SNVs located in a minuscule organelle by using conventional fluorescence imaging techniques. Fortunately, the remarkable progress made in super-resolution imaging technologies and photo-expansion microscopy present an unprecedented opportunity to visualize and distinguish those SNVs at a much higher spatial resolution level within individual cells, even within minuscule organelles. In addition, the delivery efficiency and stability of fluorophore-labeled probes are important factors for improving *in situ* SNV imaging performance, and thus should be carefully evaluated.

We anticipate there will be numerous future advancements in the SNV assay field, and we are eager to explore new opportunities within our own research group to design and apply CRISPR technology to address significant problems and requirements in this area. One particular area of interest in the future is imaging and quantification of SNVs *in vivo*. By observing and measuring these genetic variations within living organisms, we can delve deeper into the complexities of SNV-related diseases and discover new ways to diagnose and treat diseases based on individual genetic profiles. Another fascinating area of research is the potential for SNV editing to revolutionize disease treatment and prevention. With recent advancements in gene editing technology, there is growing optimism that scientists may be able to target specific genetic mutations responsible for a wide range of diseases, from cancer to inherited disorders. This could open up entirely new avenues for personalized medicine, allowing doctors to tailor treatments based on an individual's unique genetic makeup.
